# Coarse Alignment Methodology of Point Cloud Based on Camera Position/Orientation Estimation Model

**DOI:** 10.3390/jimaging9120279

**Published:** 2023-12-14

**Authors:** Suhong Yoo, Namhoon Kim

**Affiliations:** 1 Department of Drone and GIS Engineering, Namseoul University, 91, Daehak-ro, Seonghwan-eup, Seobuk-gu, Cheonan-si 31020, Republic of Korea; shyoo@nsu.ac.kr; 2 Department of Civil Engineering and Environmental Sciences, Korea Military Academy, 574, Hwarang-ro, Nowon-gu, Seoul 01805, Republic of Korea

**Keywords:** place recognition, pose estimation, mapping, sensor fusion for localization, LiDAR, coarse alignment, point cloud registration

## Abstract

This study presents a methodology for the coarse alignment of light detection and ranging (LiDAR) point clouds, which involves estimating the position and orientation of each station using the pinhole camera model and a position/orientation estimation algorithm. Ground control points are obtained using LiDAR camera images and the point clouds are obtained from the reference station. The estimated position and orientation vectors are used for point cloud registration. To evaluate the accuracy of the results, the positions of the LiDAR and the target were measured using a total station, and a comparison was carried out with the results of semi-automatic registration. The proposed methodology yielded an estimated mean LiDAR position error of 0.072 m, which was similar to the semi-automatic registration value of 0.070 m. When the point clouds of each station were registered using the estimated values, the mean registration accuracy was 0.124 m, while the semi-automatic registration accuracy was 0.072 m. The high accuracy of semi-automatic registration is due to its capability for performing both coarse alignment and refined registration. The comparison between the point cloud with refined alignment using the proposed methodology and the point-to-point distance analysis revealed that the average distance was measured at 0.0117 m. Moreover, 99% of the points exhibited distances within the range of 0.0696 m.

## 1. Introduction

### 1.1. Background

The automatic registration of point clouds is a significant research area in civil engineering, surveying, and building information modeling (BIM). To acquire extensive 3D spatial data, aerial laser scanning (ALS) or terrestrial laser scanning (TLS) methodologies utilizing light detection and ranging (LiDAR) instruments are deployed across multiple stations, necessitating the registration of survey outcomes from each station. This process is particularly crucial in indoor environments with many occluded areas, thus necessitating the use of multiple stations [1,2,3]. Point cloud registration is also necessary when using LiDAR surveying to collect data in a vast outdoor area [4].

There are two stages of automatic point cloud registration: coarse alignment and refined alignment [5]. The approximate initial values of the point cloud registration parameters are acquired using coarse alignment and are fine-tuned using refined alignment. For coarse alignment, constraints such as point, line, and surface can be used. As a representative example, the authors in [6] developed the feature-based 4-point congruent sets (F-4PCS) method and successfully performed non-target feature-based registration. The authors in [7] proposed a local feature statistics histogram (LFSH) local feature descriptor for point cloud registration that rapidly produced robust results. Image-based matching can also be affected using LiDAR and optical cameras. Ref. [8] registered point clouds by bundle adjustment using a camera attached to a LiDAR device. Ref. [9] set an initial value for the iterative closest point (ICP) using a feature detection algorithm. ICP is a representative method for refining alignment [10,11]. Men et al. [12] proposed a 4D ICP algorithm using a 3D point cloud and also a hue color domain. As such, various methods have been proposed for coarse alignment. However, according to the study in [9], there are some restrictions for automatic registration. It was necessary to have a sufficiently wide overlapped area to perform point cloud registration using targets, or to use the surveying result of a relatively small area. It is still difficult to perform automatic point cloud registration in situations where the overlapped area is not large or there is no target.

Using the position information acquired by a LiDAR may be proposed as the simplest approach for solving this problem. Since the Global Navigation Satellite System (GNSS) or Inertial Measurement Unit (IMU) is attached to a LiDAR, coarse alignment can be performed without the aid of an algorithm if accurate sensor positions can be obtained. In the study of [13], a laser scanner, GNSS, and Inertial Navigation System (INS) were used for Mobile Mapping System (MMS) point cloud registration. Ref. [14] proposed a method using a laser tracker to determine the six degrees of freedom (DOF) of a multisensory system for 3D data collection. In addition, various studies used LiDAR positional information [15,16,17]. However, in the case of indoor surveying, the use of GNSS is limited. Also, when using INS, the accuracy of the sensor directly affects the quality of the LiDAR’s position.

**Figure 1:**
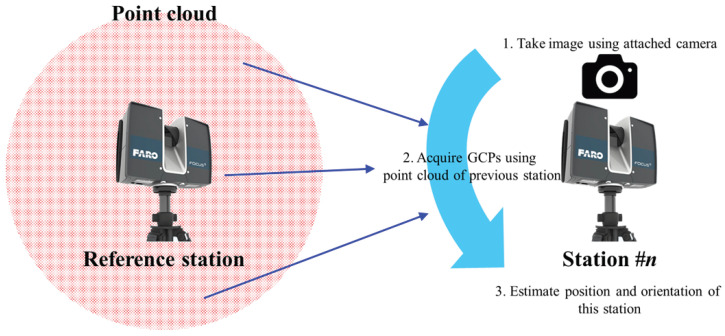
Conceptual diagram for estimating camera position and orientation.

This research presents a novel method for coarse registration based on the LiDAR device’s position and orientation, which does not rely on GNSS or an IMU coupled with a LiDAR. Instead, the LiDAR’s position and the stations’ orientations are estimated using the point cloud surveying results of the reference station, and coarse registration is performed based on the estimated value. A pinhole camera model is utilized to estimate the camera’s position and orientation. The pinhole camera model is a simplified geometric transformation that converts a three-dimensional scene into a two-dimensional image plane [18]. The key assumption in the pinhole camera model is that light travels in straight lines. Numerous studies have effectively modeled the structure of cameras with precision using this simplistic camera model. Many prior research endeavors have employed the pinhole camera model for a variety of investigations, including camera calibration and sensor position estimation [19,20,21,22,23]. Unlike existing methods that are based on the collinearity equation, the proposed method estimates the location of the laser scanning device using a single image without initial values. To test the accuracy of the suggested technique, a checkerboard marker and an automatic matching result provided by the LiDAR vendor were utilized. Figure 1 presents a conceptual diagram of this study.

In this research, our approach involved employing a pinhole camera model-based method to estimate the position of the device using a camera attached to the LiDAR. We opted not to use Single Photo Resection (SPR) due to its reliance on the initial position and orientation parameters of the LiDAR, and the Perspective-n-Point (PnP) solution due to its dependency on the calibration values of the LiDAR camera. Considering that the reliability of the estimated values with the Direct Linear Transformation (DLT) algorithm is lower compared to the pinhole camera model-based method, we ultimately conducted the estimation of the LiDAR’s position and orientation using the pinhole camera model-based method. Specifically, the pinhole camera model was selected due to its advantageous feature that allows to obtain the result value through a straightforward calculation when an adequate number of Ground Control Points (GCPs) are available.

### 1.2. Related Work

#### 1.2.1. Estimating Camera Position and Orientation

There are various methods such as SPR [24,25,26,27], the law of cosine [28], Procrustes algorithm [29,30,31], PnP algorithm [32], DLT algorithm [33], and pinhole camera-based algorithm [34,35,36] to estimate the position and orientation of the sensor using the camera image. Among these methods, SPR is a widely recognized algorithm for estimating EOPs [26,37]. It operates by iteratively adjusting three or more control points based on the collinearity equation. Efforts have been made to enhance the efficiency of SPR. Nonetheless, the reliance of SPR on accurate initial EOP values poses a challenge when such values are unspecified. The same is true for the law of cosine algorithm, Procrustes algorithm, and PnP algorithm where IOP is required. Particularly, the PnP algorithm, introduced by Fischler and Bolles [32], estimates the position and orientation of a camera using a 3D object’s corresponding point and a 2D image. Employing a perspective projection model, it is extensively utilized in fields like indoor positioning and robotics. However, these methods presuppose knowledge of the camera’s interior orientation information.

DLT model enables simultaneous estimation of the camera’s IOPs and EOPs. DLT parameters are employed to represent EOP parameters, and the least-square solution (LESS) is commonly used for their estimation. This method is widely applied in photogrammetry and computer vision due to its simplicity and the utilization of a straightforward formula for estimating the camera’s IOPs and EOPs. The advantage of using the Direct Linear Transform (DLT) in camera pose estimation lies in its ability to perform the estimation by calculating a set of simple 11 parameters based on control points. This feature has led to the widespread use of DLT in various studies for camera pose estimation [38,39,40]. However, the accuracy of the estimated IOPs and EOPs using the DLT model is lower compared to physical models like the collinearity equation or the coplanarity equation [41]. When using Direct Linear Transform (DLT) model to estimate camera pose, it must be noted that it is highly sensitive to noise, and if control points satisfy the collinear condition, it may fail to perform accurate pose estimation. Additionally, it has the drawback of not satisfying the regular orthogonality properties for the rotation matrix. Furthermore, in the case of affine projection, some information about the rotation matrix is missing, making it impossible to calculate the camera orientation [42,43].

The pinhole camera model also yields satisfactory results for EOP estimation. In certain cases, the perspective projection model has been employed to estimate radial distortion values, principal distances, and EOPs. The solution can be obtained using techniques such as the Gröbner basis or the Sylvester matrix [34,44]. The pinhole camera model-based algorithm can stably estimate the position and orientation of the camera sensor [45]. Several investigations have demonstrated the potential application of this approach to rolling shutter pose estimation as well [46,47].

#### 1.2.2. Point Cloud Registration

Numerous attempts have been made to develop an effective technique for indoor point cloud registration. Tsai and Huang [48] proposed a registration method to align a multi-view point cloud using a camera calibration technique. The authors used the pan-tilt records of the camera and transformation matrices to merge point clouds. The presented method was compared with the fast global registration method and Super4PCS and showed superiority in terms of the root mean square (RMS) results and processing time. This method was suitable to be applied when the LiDAR device rotates at one location and performs registration after acquiring a point cloud. Zhang et al. [49] proposed an end-to-end registration network (SLORNet) to overcome the difficulties in matching low-density point clouds with a small overlap. This method performed exceptionally well for indoor point cloud registration but has limitations for outdoor registration. A robust ICP (RICP) method for the registration of Red, Green, Blue, and Depth (RGB-D) point clouds was also proposed [50]. In this study, the existing ICP algorithm was improved regarding region selection, point matching, and noise treatment. In particular, silent object detection (SOD), which is based on a deeply supervised network was used to search for matching points and produced satisfactory indoor point cloud registration. Various algorithms have been used to register indoor point clouds. These methods prove efficient when the point cloud acquisition point is at a short distance, e.g., when matching the point cloud of one room. When registering the point cloud of a long corridor with many corners and when the exact location of the LiDAR cannot be specified because it is indoors, registration must be performed using another method.

Extensive research has been conducted on feature-based registration algorithms as well. You et al. [51] proposed a point cloud registration algorithm based on the 3D Neighborhood Point Feature Histogram (3DNPFH) descriptor. Their algorithm involves uniformly sampling the point cloud to extract key points, transferring these key points to a new 3D coordinate system by constructing a local reference coordinate system, and reducing the coordinate search space during feature matching by focusing on key points of similar surfaces in close proximity. They constructed a neighborhood point feature histogram by combining density, curvature, and normal vector information to find precise matches. The authors highlighted in their paper that the algorithm presented achieves faster registration compared to existing methods. In a study by Li et al. [52], an improved Whale Optimization Algorithm (IWOA) and an improved ICP (Iterative Closest Point)-based registration algorithm were proposed to address the issues of low accuracy and efficiency in point cloud registration for stereo camera systems. This study also employed a two-step approach involving coarse registration and refined registration for point cloud registration. The proposed algorithms in this paper also demonstrated improved registration accuracy and speed. However, it should be noted that the sample point clouds used in both studies were Stanford point clouds, and experiments were not conducted on continuously acquired point clouds while the LiDAR was in motion.

Alicandro et al. [53], Xiong et al. [54], and Liu et al. [55] conducted research on feature-based registration algorithm targeting indoor and outdoor architectural structures. Alicandro et al. [53] conducted research focused on fine registration process for large-scale point clouds. Their proposed method introduces a novel approach that utilizes planar approximations of geometric features associated with roof structures (PARF). The PARF method demonstrates superior resilience to noise in comparison to other analysis techniques, ensuring more reliable results even in the presence of noisy data. Additionally, it exhibits notable computational efficiency, allowing for faster processing and analysis of point cloud data. Moreover, it offers the advantage of enabling point cloud registration in dynamic environments, surpassing the efficiency of the conventional ICP method. When compared the results with GNSS surveying, marker-based registration showed an error range of 0.011–0.012%, while the PARF methodology exhibited a lower positional error of 0.004–0.005%. In terms of computational time, the ICP algorithm required 120 min, whereas PARF only took 30 min, thereby demonstrating its superiority in this aspect as well.

Xiong et al. [54] proposed a point cloud registration algorithm based on Gaussian-weighting projected image matching. This method demonstrated its strength in registering point clouds acquired from multiple stations. Firstly, the point cloud was normalized into a 2D grid, where the point density of each grid cell was normalized using a Gaussian weighting function. Secondly, the SIFT (scale-invariant features) algorithm was employed for image matching, and a line segment endpoint verification method was used to filter out incorrect matches. Lastly, the transformation matrix between adjacent stations’ point clouds was calculated based on image matching. The method was reported to have an operation time 4 to 10 times faster and a registration accuracy 2 to 6 times higher compared to the conventional 4PCS method. However, as acknowledged by the authors, a limitation of this approach is that it is applicable only to point clouds with planar surfaces.

Liu et al. [55] introduced a methodology for registering the point cloud of an irregular structure (specifically, a dome) in indoor environments. The methodology comprises three blocks: coarse registration, partial fine registration, and full fine registration. In this study, the coarse registration step utilized the image-based coarse registration method proposed by Manush et al. [56]. Both studies utilized a LiDAR system known as Stockpile Monitoring and Reporting Technology (SMART) to measure the volume of indoor stockpiles. The coarse registration step consisted of six stages. Firstly, feature points between the reference station’s acquired image and images acquired from other stations were detected using the SIFT algorithm. After removing lens distortion, stereo-pair selection was performed, and the rotation angle of the camera was estimated. After selecting the rotation matrix and features, an iterative process was employed to fine-tune the camera rotation angle. This refinement step resulted in excellent outcomes even when registering sparse point clouds, showcasing the method’s effectiveness for both regular and irregular structures. The system has demonstrated the capability to generate volume estimates that closely resemble those obtained from Terrestrial Laser Scanner (TLS), with a difference within the range of 1%.

Despite the abundance of research in point cloud registration, indoor environments present additional challenges that make the task more difficult. Registration and localization using indoor point clouds can encounter obstacles such as self-similarity within the environment and the presence of unforeseen objects [57]. In Mahmood et al.’s study, they addressed these challenges by proposing geometric feature-based localization and registration techniques to overcome these difficulties. In the study conducted by Luo et al. [58], challenges were encountered during the registration of indoor point clouds obtained from TLS and Mobile Laser Scanner (MLS) systems. To address this issue, the researchers leveraged existing architectural reference data as a means to overcome the difficulties associated with indoor point cloud registration.

#### 1.2.3. Position and Orientation Estimation of Sensors Using Point Cloud

In Baek et al.’s study [59], authors focused on the position of the point cloud acquisition platform (mobile LiDAR) in underground spaces. The authors utilized fast point feature histograms to search for point features in both local and global point clouds and employed random sample consensus (RANSAC) and ICP for registration. They then estimated the position of the mobile LiDAR using these techniques. While their study shares similarities with ours, there are clear differences. The main distinguishing factor is whether a pre-existing global point cloud is available or not. Additionally, it is important to consider whether the estimation is limited to the position of the platform alone or includes the estimation of orientation as well.

In the study conducted by Wasik et al. [60], they utilized 2D point cloud data obtained from other robots to achieve relative positioning of closely-grounded robots. By employing a simple circle fitting algorithm on the 2D point cloud, the local coordinate frame of the robot of interest could be estimated. The objective of this research was to determine the position of robots of the same type in close proximity.

Salles et al. [61] conducted a study focused on aerial point clouds. They employed the Normalized Cross-Correlation method to match the trajectory point cloud with a pre-existing reference database. In this study, the accuracy of estimation was enhanced by utilizing mapping data that integrates terrain, canopy top, and intensity information. By applying a 2D transformation called binning, they successfully tracked the trajectory of the unmanned aerial vehicle (UAV).

In Jiang et al.’s study [62], they proposed a framework for estimating the position and attitude of a robot using IMU (Inertial Measurement Unit) and LiDAR data. The Rank Kalman filter was employed in their approach. Through experiments conducted on small-scale trajectories, the authors demonstrated that their proposed method improved the accuracy of robot movement path estimation by 23.84% and 25.26% in the X and Y axis directions, respectively. This study also aimed to track the two-dimensional motion of the robot. Indeed, a significant number of studies have been dedicated to extracting the sensor’s position from a pre-existing point cloud. Positioning using point cloud has been studied a lot in the field of position estimation of mobile systems such as SLAM and ROS, and it was confirmed that the majority of the target data were 2D point cloud.

In summary, as observed from Section 1.2.1, Section 1.2.2 and Section 1.2.3, there are numerous prior studies on sensor position/orientation using 2D image pixel coordinates or 3D point cloud coordinates estimation and point cloud registration. However, despite the existence of many previous research efforts, it has been challenging to find studies specifically focusing on performing coarse registration of point clouds using the estimation of LiDAR’s position and orientation. Although imaging sensors have been attached to LiDAR, they have mainly been utilized to provide RGB information to the point cloud without additional functionalities. Additionally, while there have been numerous studies on camera pose estimation, research on integrating camera pose estimation with other sensors has been limited. Therefore, in this paper, we conducted research on a methodology for registering 3D point clouds by integrating the comprehensive theories presented. We focused on estimating the position and orientation of the point cloud acquisition platform using the imaging sensor attached to LiDAR and performed coarse registration. The method proposed in this study proves particularly beneficial in scenarios where determining the position and orientation of LiDAR is challenging, such as in underground or indoor environments. Moreover, it offers the advantage of performing point cloud registration outdoors using the same methodology. Additionally, since it relies on estimating the position and orientation of LiDAR using a two-dimensional image, it exhibits strengths in terms of computational requirements and processing time. Lastly, a noteworthy advantage is its capability to conduct coarse registration irrespective of the distance between point clouds.

## 2. Methodology

### 2.1. Camera Geometric Model and Estimating Position/Orientation Method

#### 2.1.1. Pinhole Camera Model

The basic pinhole camera model is expressed using Equation (1) [63].

(1) $$\lambda x_{I}=PX_{W}\;$$

where scalar λ is a scale factor, the vector $x_{I}$ is the homogeneous vector of the image point, $X_{W}$ is the homogeneous vector of the object point, and *P* is the 3 × 4 homogeneous camera projection matrix. When considering the application of principal point offset, pixel ratio, and skew, Equation (1) is modified into Equation (2) as follows.

(2) $$\left[\begin{array}{c} fX+Zp_{x}\\ fY+Zp_{y}\\ Z \end{array}\right]=\left[\begin{array}{cccc} \alpha f & s & p_{x} & 0\\ 0 & \beta f & p_{y} & 0\\ 0 & 0 & 1 & 0 \end{array}\right]\left[\begin{array}{c} X\\ Y\\ Z\\ 1 \end{array}\right]$$

where *f* is the focal length, $p_{x}$ and $p_{y}$ are the locations of the principal point, α and β are the pixel ratios, and *s* is the skew parameter.

**Figure 2:**
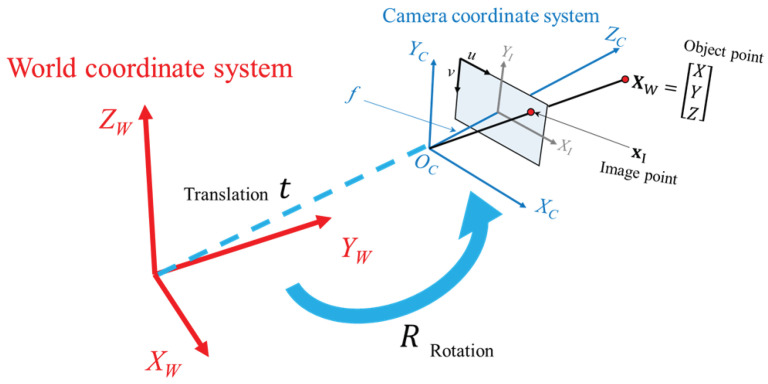
World coordinate system and camera coordinate system.

Figure 2 illustrates the process of transforming between the world coordinate system and the camera coordinate system. This transformation involves the utilization of rotation (*R*) and translation (t) operations. The geometric camera model, incorporating camera rotation and translation, is applied in the following Equation (3).

(3) $$\lambda x_{I}=PX_{W}=K\left[R|t\right]X_{W}$$

where K=diag(1,1,w) is the camera calibration matrix for $w=\frac{1}{f}$.

#### 2.1.2. Absolute Position/Orientation Estimation and Calibration Using Pinhole Camera Model

In general, since the LiDAR’s important data are a 3D point cloud, the camera attached to a LiDAR is only used to acquire the RGB value of the point cloud. Therefore, the provided camera’s IOPs are bound to be limited. In particular, the radial distortion parameter, as well as the camera’s focal length information, are not given in most cases. Therefore, in this study, a method is used that simultaneously calibrates the camera and estimates the absolute position/orientation. For this, assume that the camera’s principal point is located in the center of the image, the skew parameter is 0, and the pixel ratio is 1. This is consistent with the general characteristics of modern cameras [44,64,65,66]. Under these assumptions, a process for camera calibration and position/orientation estimation was proposed [34,36,44,67]. The image coordinates of the radially distorted image can be expressed as Equation (4).

(4) $$x_{I,i}={\left[\begin{array}{ccc} x_{i} & y_{i} & 1+k_{1}\left(x_{i}^{2}+y_{i}^{2}\right)+k_{2}{\left(x_{i}^{2}+y_{i}^{2}\right)}^{2}+k_{3}{\left(x_{i}^{2}+y_{i}^{2}\right)}^{3} \end{array}\right]}^{T}$$

where $x_{I,i}$ is the $i^{th}$ image point coordinates; $k_{1},k_{2},$ and $k_{3}$ are radial distortion parameters. Let us define the skew–symmetric matrix of vector a as the following Equation (5).

(5) $${\left[a\right]}_{\times }=\left[\begin{array}{ccc} 0 & -a_{3} & a_{2}\\ a_{3} & 0 & -a_{1}\\ -a_{2} & a_{1} & 0 \end{array}\right]$$

The following Equation (6) can be obtained by multiplying both sides of the pinhole camera model by the skew–symmetric matrix of vector $x_{I,i}$.

(6) $$\left[\begin{array}{ccc} 0 & -(1+k_{1}r_{i}^{2}+k_{2}r_{i}^{4}+k_{3}r_{i}^{6}) & y_{i}\\ 1+k_{1}r_{i}^{2}+k_{2}r_{i}^{4}+k_{3}r_{i}^{6} & 0 & -x_{i}\\ -y_{i} & x_{i} & 0 \end{array}\right]\left[\begin{array}{cccc} p_{11} & p_{12} & p_{13} & p_{14}\\ p_{21} & p_{22} & p_{23} & p_{24}\\ p_{31} & p_{32} & p_{33} & p_{34} \end{array}\right]\left[\begin{array}{c} X_{i}\\ Y_{i}\\ Z_{i}\\ 1 \end{array}\right]=0$$

The third row of Equation (6) can be written as the following Equation (7):

(7) $$-y_{i}\left(p_{11}X_{i}+p_{12}Y_{i}+p_{13}Z_{i}+p_{14}\right)+x_{i}\left(p_{21}X_{i}+p_{22}Y_{i}+p_{23}Z_{i}+p_{24}\right)=0$$

If seven GCPs are available, Equation (7) can be transformed into a matrix form as shown in Equation (8).

(8) $$Ax=\left[\begin{array}{cccccccc} -y_{1}X_{1} & -y_{1}Y_{1} & -y_{1}Z_{1} & -y_{1} & -x_{1}X_{1} & -x_{1}Y_{1} & -x_{1}Z_{1} & x_{1}\\ -y_{2}X_{2} & -y_{2}Y_{2} & -y_{2}Z_{2} & -y_{2} & -x_{2}X_{2} & -x_{2}Y_{2} & -x_{2}Z_{2} & x_{2}\\ \vdots & \vdots & \vdots & \vdots & \vdots & \vdots & \vdots & \vdots \\ -y_{7}X_{7} & -y_{7}Y_{7} & -y_{7}Z_{7} & -y_{7} & -x_{7}X_{7} & -x_{7}Y_{7} & -x_{7}Z_{7} & x_{7} \end{array}\right]\left[\begin{array}{c} p_{11}\\ p_{12}\\ p_{13}\\ p_{14}\\ p_{21}\\ p_{22}\\ p_{23}\\ p_{24} \end{array}\right]=0$$

Using Singular Value Decomposition (SVD), the value of $p_{11}$ to $p_{24}$ can be obtained. When decomposing matrix *A* into *U*, Σ, and *V*, the optimal solution for minimizing |Ax|, which corresponds to finding the solution for Ax=0, is obtained using the right singular vector of the V matrix. It is important to note that the magnitude of this right singular vector is 1. However, further calibration or normalization steps may be necessary to ensure the desired scale or magnitude of the solution. The scale value can be corrected using the property of the rotation matrix. Although the number of GCPs can be reduced by using methods such as Gröbner basis or Sylvester matrix [34,35,44]. However, the elements of the matrix *P* can be obtained using 7 GCPs and the characteristic of the rotation matrix to simplify the algorithm [45].

Let us define the $P^{\prime }$ matrix as the following Equation (9).

(9) $$P^{\prime }=\left[\begin{array}{ccc} p_{11} & p_{12} & p_{13}\\ p_{21} & p_{22} & p_{23}\\ p_{31} & p_{32} & p_{33} \end{array}\right]=KR$$

Since the matrix *K* is a diagonal constraint matrix and *R* is the rotation matrix, the three rows of the matrix $P^{\prime }$ are perpendicular. Also, the norm of the first and second-row vectors are the same. Therefore, Equation (10) can be established.

(10) $$\begin{array}{c} p_{11}p_{21}+p_{12}p_{22}+p_{13}p_{23}=0\\ p_{31}p_{11}+p_{32}p_{12}+p_{33}p_{13}=0\\ p_{31}p_{21}+p_{32}p_{22}+p_{33}p_{23}=0\\ p_{11}^{2}+p_{12}^{2}+p_{13}^{2}-p_{21}^{2}-p_{22}^{2}-p_{23}^{2}=0 \end{array}$$

In Equation (10), $p_{32}$ and $p_{33}$ can be expressed using $p_{31}$ as the following Equation (11).

(11) $$p_{32}=-\frac{p_{31}\left(p_{11}p_{23}-p_{21}p_{13}\right)}{p_{12}p_{23}-p_{22}p_{13}}=p_{31}c_{1},p_{33}=-\frac{p_{31}\left(p_{21}p_{12}-p_{11}p_{22}\right)}{p_{12}p_{23}-p_{22}p_{13}}=p_{31}c_{2}$$

The remaining five unknown parameters are $p_{31}$, $p_{34}$, $k_{1}$, $k_{2}$, and $k_{3}$. Using the second row of Equation (7), these unknowns can be calculated.

(12) $$Mv_{\mathrm{unknown}}=v_{\mathrm{observation}}$$

where

(13) $$M=\begin{array}{cc} \; & \left[\begin{array}{ccc} x_{i}X_{i}+c_{1}x_{i}Y_{i}+c_{2}x_{i}Z_{i} & x_{i} & -r_{i}^{2}\left(p_{11}X_{i}+p_{12}Y_{i}+p_{13}Y_{i}+p_{14}\right) \end{array}\right.\\ \; & \;\;{\left.\begin{array}{cc} -r_{i}^{4}\left(p_{11}X_{i}+p_{12}Y_{i}+p_{13}Y_{i}+p_{14}\right) & -r_{i}^{6}\left(p_{11}X_{i}+p_{12}Y_{i}+p_{13}Y_{i}+p_{14}\right) \end{array}\right]}_{i\times 5} \end{array}$$

(14) $$v_{\mathrm{unknown}}={\left[\begin{array}{ccccc} p_{31} & p_{34} & k_{1} & k_{2} & k_{3} \end{array}\right]}_{5\times 1}^{T}$$

(15) $$v_{\mathrm{observation}}={\left[\begin{array}{c} p_{11}X_{i}+p_{12}Y_{i}+p_{13}Z_{i}+p_{14} \end{array}\right]}_{i\times 1}$$

By applying the least-squares solution (LESS), the values of all elements of the $v_{\mathrm{unknown}}$ can be obtained. Finally, the radial distortion parameter, camera position, and rotation can be obtained. The last unknown focal length can be obtained using the first and last rows of matrix P as the following Equations (16) and (17).

(16) $$w^{2}p_{11}^{2}+w^{2}p_{12}^{2}+w^{2}p_{13}^{2}-p_{31}^{2}-p_{32}^{2}-p_{33}^{2}=0$$

(17) $$f=\frac{1}{w}=\sqrt{\frac{p_{11}^{2}+p_{12}^{2}+p_{13}^{2}}{p_{31}^{2}+p_{32}^{2}+p_{33}^{2}}}$$

### 2.2. Data Acquisition

#### 2.2.1. Sensors

The experiment conducted in this study employed the Leica BLK360 LiDAR; the technical specifications of this device, as provided by Leica Geosystems, Seoul, Korea, are presented in Table 1.

**Figure 3:**
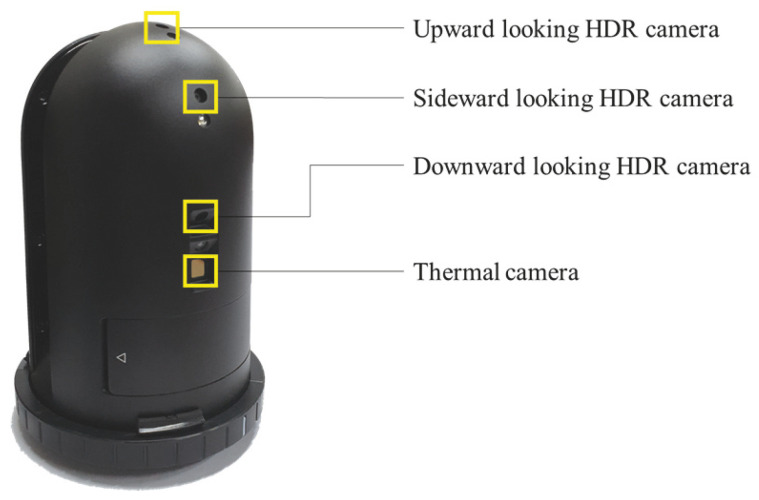
Leica BLK360 LiDAR attached cameras.

**Figure 4:**
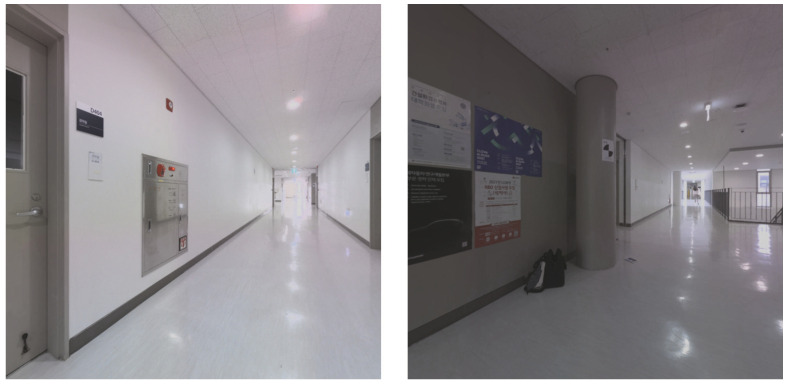
Images captured by LiDAR cameras.

BLK360 is a terrestrial laser scanner with a camera attached, and it acquires 6 images in 4 directions. The Leica BLK360 is equipped with three cameras that capture upward, sideward, and downward images, allowing for the creation of 360° × 300° dome images. For this study, mainly 4 sideward images were utilized. The process of acquiring side images involves rotating the LiDAR at intervals of 90° and capturing images using the sideward camera. Figure 3 depicts the cameras that are mounted on the LiDAR system, while Figure 4 illustrates 2 sample images that were captured by these cameras. The BLK360 device was chosen for this study due to its user-friendly nature, allowing for easy utilization of the images captured from the camera attached to the LiDAR. This facilitated a straightforward comparison with the proposed methodology by enabling simple measurements and automatic registration. Furthermore, the proposed methodology can also be applied for coarse alignment when using TLS with an attached camera, and thus not limited to the BLK360 device.

**Figure 5:**
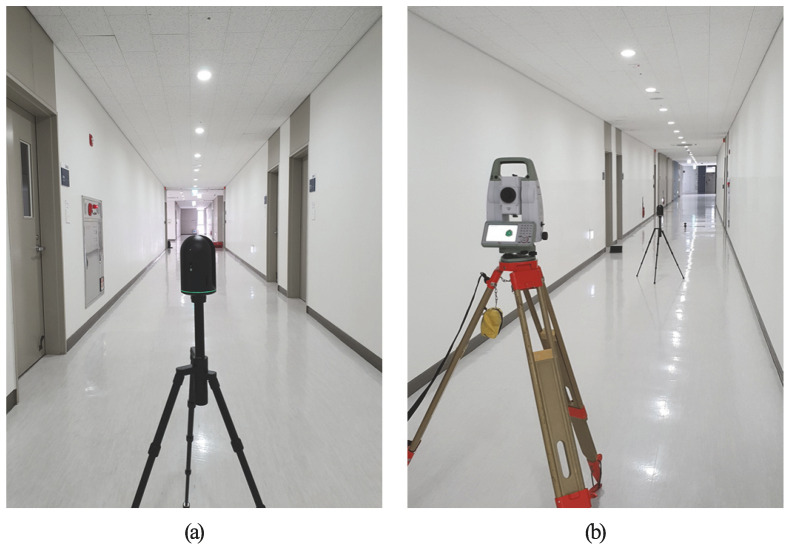
Experiment using surveying equipment: (**a**) Acquired point clouds using Leica BLK360 LiDAR, (**b**) surveying the position of LiDAR stations using TS13 total station.

**Figure 6:**
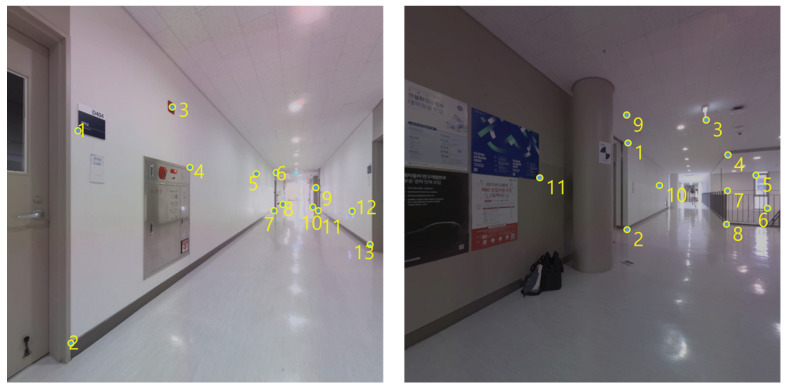
Feature point for GCP.

The accurate location of each station was obtained through the application of Leica TS13 total station. Figure 5 displays the implementation of LiDAR surveying and LiDAR position surveying in conjunction with the total station. To implement the methodology proposed in this study, it is necessary to identify feature points in images that can serve as GCPs. For each station image, a selection of 9 to 12 GCPs was made. Figure 6 provides an illustrative example of a chosen GCP.

#### 2.2.2. Test Site

**Figure 7:**
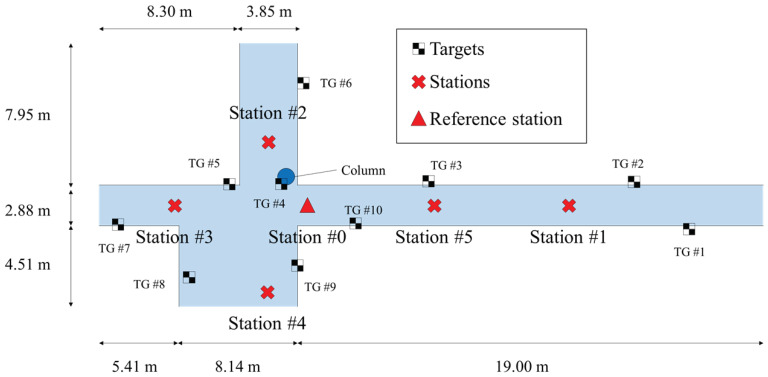
The layout of LiDAR stations.

The suggested process was verified through an indoor LiDAR survey conducted in Engineering Hall 4 at Yonsei University. The layout of the test site and the location of each station are shown in Figure 7. The target area, which consists of long corridors and various obstacles, was suitable for verifying the proposed process. At the reference station, a point cloud was acquired through laser scanning and used to acquire the GCPs for the remaining stations. After extracting the GCPs from the acquired point cloud, the position of each station was estimated using the suggested methodology. To determine the registration accuracy, black and white targets were placed in the test site as shown in Figure 7. The red triangle represents the reference station, which is Station 0. The red X marks represent the various stations where point clouds and images were acquired. The black and white checkered pattern denotes the targets that were attached for accuracy comparison purposes.

## 3. Results and Discussions

### 3.1. Point Cloud of Each Station

**Figure 8:**
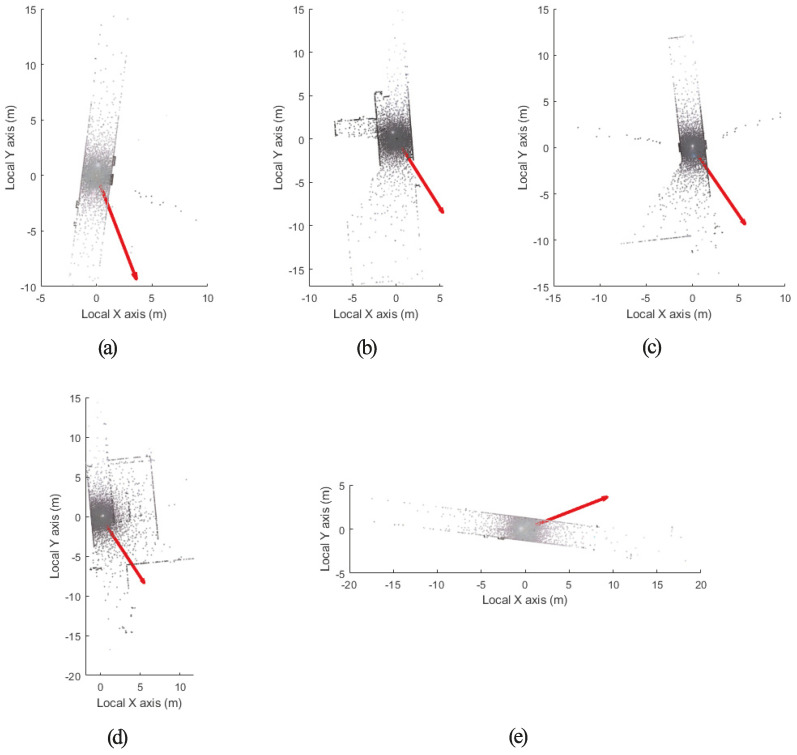
Point clouds and local camera orientation vectors: (**a**) Station 1, (**b**) Station 2, (**c**) Station 3, (**d**) Station 4, (**e**) Station 5.

Figure 8 shows the point clouds acquired from each station and the camera’s view direction of each station. Since no GPS or IMU was applied, each camera center was set to the origin. The camera view direction was estimated using the image and point cloud of each station. In this study, the camera view direction estimated using the point cloud of each station was designated as a local camera orientation vector.

### 3.2. LiDAR Position and Orientation Estimation

The position and orientation of the LiDAR were estimated by utilizing the point cloud data from reference station and the images from each station. The world camera orientation was determined based on the estimated orientation obtained from the point cloud data of reference station. For each station, 9 to 12 GCPs were obtained by utilizing the images captured at the respective station and the reference point cloud gathered at reference station. The point cloud data were rotated using the local camera orientation vector and the world camera orientation vector obtained from the previous subsection and translated using the estimated LiDAR position. In the presented methodology, while only 7 GCPs are technically required, a selection of 9 to 12 GCPs was made to minimize estimation errors and eliminate outliers. The C(n,7) estimation process was employed, followed by the removal of estimated outlier values using the RANSAC algorithm. From the remaining candidate solutions, the estimation result with the lowest reprojection error was considered as the position and orientation of the LiDAR device. Table 2 summarizes the translation vectors, the local camera orientation vectors, and the world camera orientation vectors of each station. Only the lever-arm vector representing the exact position of the camera in the point cloud coordinate system was calculated for reference station. Since the point cloud of reference station was used as a reference point cloud, point cloud rotation was not required and therefore, the boresight vector was not calculated.

This study examined the precision of both the position and orientation of the LiDAR. The position value of the LiDAR, as measured by the total station, was considered the true value for the target coordinates. The precision of the LiDAR’s orientation was evaluated indirectly using the reprojection errors of checkpoints that were acquired separately from the GCPs used for estimating the LiDAR’s position with SPR. Table 3 presents a summary of the position errors and reprojection errors for each station.

The estimated position error of the stations averaged 0.072 m with a maximum of 0.115 m. The estimated position error of semi-automatic registration averaged 0.070 m with a maximum of 0.128 m. The position estimation results of the coarse alignment method proposed in this study and of semi-automatic registration, were almost identical. Additionally, the reprojection error of the proposed method averaged 2.93 pixels with 4.1 pixels as the maximum. It can, therefore, be concluded that the proposed coarse alignment method reasonably estimates the position and orientation of the LiDAR.

### 3.3. Point Cloud Registration

The acquired point clouds from the stations were subject to rotation and translation using local and world camera orientation vectors to achieve coarse alignment. Furthermore, the precision of the proposed coarse alignment technique was evaluated by comparing the registration accuracy using 10 black and white targets. The targets’ center coordinates were determined using a total station, extracted from the coarse registered and semi-automatic registered point clouds, and compared to the total station surveying outcome to calculate the registration error. Table 4 illustrates the coarse alignment error and semi-automatic error, where the proposed method exhibited a mean registration error of 0.124 m, while semi-automatic registration achieved an error of 0.072 m. Since the point cloud’s rotation contains an error, the coarse alignment method is deemed to have a higher error of approximately 0.05 m compared to semi-automatic registration. Additionally, the semi-automatic registration process involves visual confirmation or user experience to achieve coarse alignment, followed by refined alignment for the final output. In the semi-automatic method, a skilled individual with knowledge of the surveying device manually performs coarse alignment by locating the station, followed by refined alignment through software using techniques like the ICP algorithm. As a result, the semi-automatic method generally exhibits small registration errors for most targets. However, it is worth noting that the methodology presented in this study also yields registration errors that are not significantly large. Therefore, it is expected that incorporating various refined alignment algorithms into the proposed coarse alignment method would increase registration accuracy. The refined registration, utilizing the overlapping regions of each point cloud of stations, resulted in a total point cloud with alignment errors comparable to those of the semi-automatic approach. The minimum error was 0.008 m, the maximum error was 0.097 m, and the average registration error concerning the target was 0.050 m. This showed a difference of 0.022 m compared to the use of the semi-automatic methodology. The size of overlapping regions between point clouds affects the registration accuracy in the case of the ICP algorithm [68]. The point clouds utilized in this study had limited overlap due to the distinctive features of the research area, consequently influencing the final accuracy.

**Figure 9:**
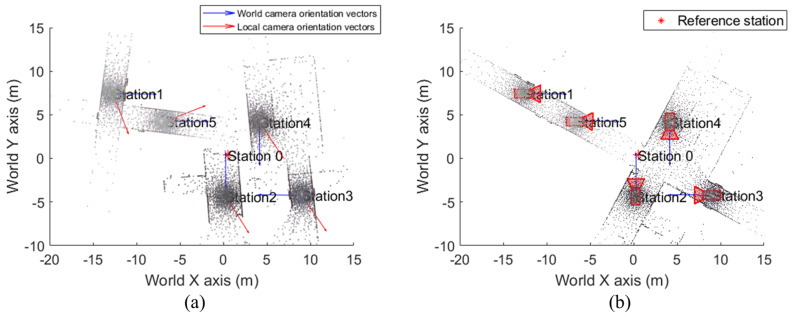
Registration results: (**a**) only translation applied, (**b**) translation and rotation applied.

Figure 9 shows the registered point cloud, while Figure 9a shows a point cloud where only translation was performed. The local camera orientation vector did not match the world camera orientation vector, and the point cloud was not properly registered. Figure 9b shows a point cloud that performed both translation and rotation. This confirms that registration was similar to that of the actual test site.

**Figure 10:**
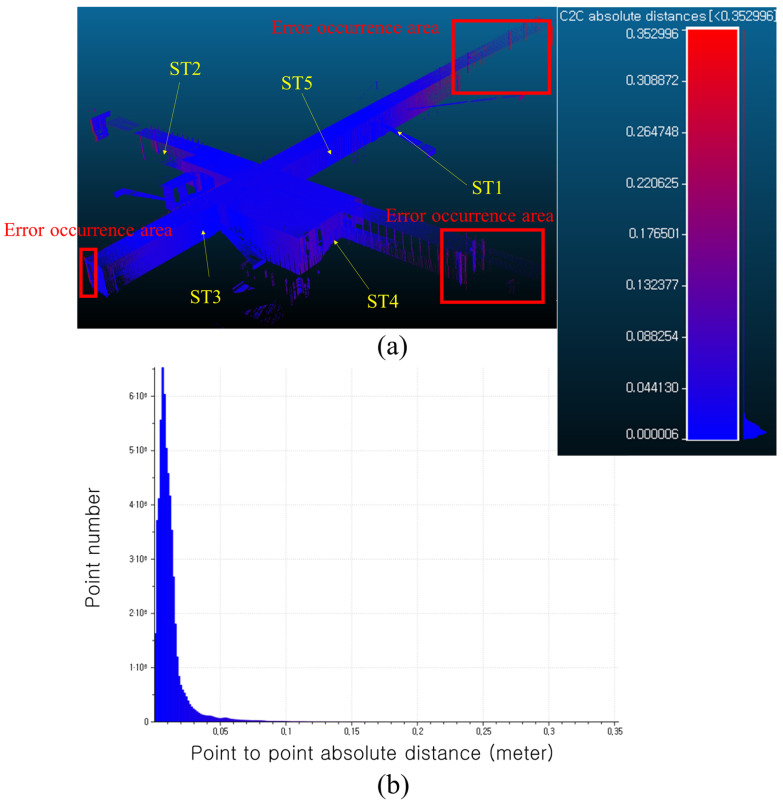
Point to point absolute distance comparison: (**a**) visualization of point to point comparison, (**b**) histogram of point to point absolute distance.

Figure 10 compares the point cloud obtained through manual alignment followed by fine alignment using the ICP algorithm with the point cloud aligned through the proposed methodology. Figure 10 illustrates the outcomes analyzed and visualized through CloudCompare V2. The numerical analysis is based on the point-to-point absolute distance to assess the differences between the two point clouds. Figure 10a shows the point-to-point absolute distance using a blue-to-red gradient, while Figure 10b presents the corresponding histogram of the results. The approximate positions of each station are indicated by yellow arrows in Figure 10a. The average distance between the 57,971,263 points is 0.0117 m, with a maximum distance of 0.3529 m. Additionally, 99% of the points have distances within 0.0696 m. Regions with larger point-to-point absolute distances, indicating higher registration errors, are predominantly observed at the ends of long corridors, as depicted by the red box in Figure 10a. This phenomenon arises due to the rotation and translation-based registration approach employed in the proposed methodology. As the distance from the station increases, errors in rotation angles have a greater impact on the positional error of the points. Consequently, larger errors are observed when moving away from stations 1, 2, 3, and 4. However, most of the errors are not excessive, and the addition of refined alignment to the proposed coarse alignment methodology can effectively mitigate these errors.

**Figure 11:**
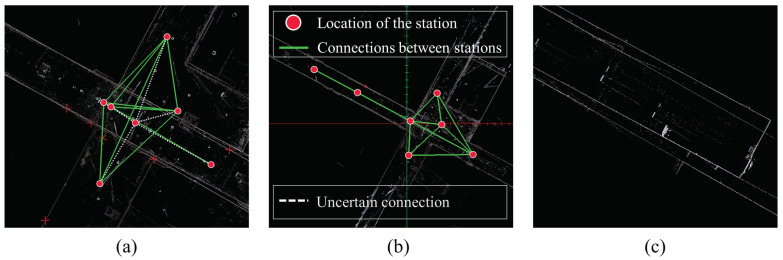
Automatic and manual registration results: (**a**) automatic links, (**b**) manual links, (**c**) point cloud incorrectly registered through automatic registration.

**Figure 12:**
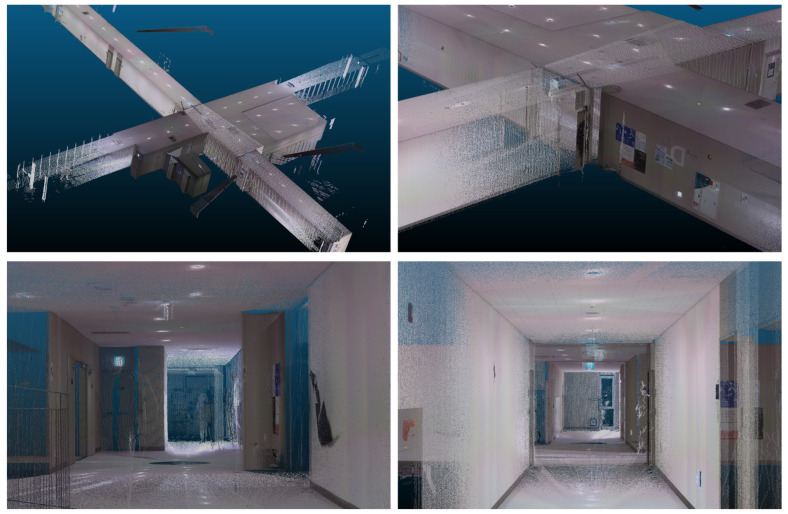
Detailed view of the registered point cloud using the proposed methodology.

The semi-automatic registration function of the BLK360 produced accurate results. However, automatic registration was not performed with long distances between stations or with insufficient overlaps even though the BLK360 supports automatic registration. In this experiment, even though additional stations were installed to facilitate automatic registration, fully automatic registration was not performed. Figure 11 shows the links and point cloud when automatic registration is performed. The program used is Leica Cyclone Register 360 (BLK Edition). Figure 11a shows the link between each station as a line, and Figure 11b shows the result of manual linking. It can be seen that the two link results are very different from each other. Figure 11c shows the point cloud using the link in Figure 11a. Specifically, Figure 11c indicates that there is an improper alignment between the long hallway and the wall. This phenomenon can be attributed to the spatial characteristics of the corridors, which often result in multiple point clouds with similar shapes. Due to the similarity in wall structures within the corridors, the ICP algorithm tends to minimize the distance between the point clouds during the matching process, leading to a potential reduction in errors. However, this characteristic of the ICP algorithm can also result in misaligned stations, as illustrated in Figure 11a. The user had to perform manual registration based on the experience at the time of the surveying to create an accurate link as shown in Figure 11b. If the method proposed in this study is used, the accurate initial value could be set to create a link for automatic registration without requiring survey experience. In contrast, when employing the method proposed in this study for point cloud registration, we observed that the misplacement of stations did not occur. The detailed appearance of the point cloud matched by the proposed method is depicted in Figure 12. Although there may be errors in the fine details such as signs due to it being a coarse alignment stage, the corridors and walls are well-matched, even in the absence of fine registration.

## 4. Conclusions

This study proposed a new methodology for coarsely registering point clouds using reference point clouds and LiDAR camera images. The positions and orientations of the LiDAR stations were established using a camera position/orientation estimation algorithm based on a pinhole camera model and point cloud registration was performed using the estimated position and orientation. To evaluate the accuracy of the proposed methodology, the position and orientation estimation results and point cloud registration accuracy were compared with the total station surveying results, which were set as true values. The reprojection errors of the checkpoints were used to compare the orientation accuracy. To calculate the registration error of the point cloud, black and white targets were attached to the research area, and their coordinates were obtained by measuring the center of the target with the total station. The results of semi-automatic registration using the registration software provided by the vendor were also compared.

Compared to the total station surveying results, the position estimation of the proposed methodology showed a mean error of 0.07 m, while semi-automatic registration showed a mean error of 0.072 m. The orientation error showed a mean of 2.93 pixels, indicating that the estimated value was reliable. The point cloud registration error was 0.124 m for the proposed methodology and 0.072 m the for semi-automatic registration. Since semi-automatic registration performs both coarse and refined alignment, it is expected that the registration error would decrease. The proposed methodology was found to be effective in calculating the initial value for refined alignment, and accurate point cloud registration could be achieved with additional point cloud adjustment. The creation of links between stations during semi-automatic registration was found to be a challenge. Nonetheless, the adoption of the methodology outlined in this study should lead to a streamlined registration procedure.

The method proposed in this study offers a straightforward approach for coarse alignment of point clouds, especially in scenarios where measuring the position of LiDAR is challenging. It can be particularly valuable for point cloud registration in indoor or underground environments where GNSS usage is impractical. In spaces characterized by numerous narrow corridors, even automatic registration methods struggle to achieve satisfactory coarse alignment. The proposed method provides the advantage of conducting point cloud registration outdoors using the same methodology. If both the image attached to the LiDAR and the reference point cloud are available, registration can be achieved regardless of the environment, whether indoor or outdoor. Furthermore, this registration approach is not limited to specific platforms and can also be applied to the outcomes of aerial LiDAR surveys. The proposed method utilizing a two-dimensional image offers several advantages. It incurs lower registration costs compared to methods employing three-dimensional point clouds. Even in scenarios where point clouds from multiple stations fail to overlap due to various factors, registration becomes feasible as long as an image capturing the reference station is available. Lastly, a notable benefit is its ability to perform coarse registration regardless of the distance between point clouds. By combining the method proposed in this study with various feature point searching algorithms, it is anticipated that efficient measurements can be conducted, thereby further enhancing the alignment process.

## Figures and Tables

**Table 1:** Specifications of BLK360.

Dimensions	Height: 165 mm/Diameter: 100 mm
Distance measurement system	High-speed time-of-flight enhanced by Waveform Digitizing (WFD) technology
Laser class	1 (in accordance with per IEC 60825-1:2014)
Wavelength	830 nm
Field of view	360° (horizontal)/300° (vertical)
Range	min. 0.6–up to 60 m
Point measurement rate	up to 360,000 pts/s
Ranging accuracy	4 mm @ 10 m/7 mm @ 20 m
Camera System	15 Mpix 3-camera system, 150 Mpx full dome capture, HDR, LED flash Calibrated spherical image, 360° × 300°
Thermal Camera	FLIR technology based longwave infrared camera Thermal panoramic image, 360° × 70°

**Table 2:** Local camera orientation vectors and world camera orientation vectors.

Station Number	Translation Vector			Local Camera Orientation Vector			World Camera Orientation Vector		
	X (m)	Y (m)	Z (m)	X (m)	Y (m)	Z (m)	X (m)	Y (m)	Z (m)
Reference station	0.489	0.387	0.002	-	-	-	-	-	-
Station 1	−12.715	7.464	0.021	0.359	−0.934	−0.005	0.999	−0.017	−0.002
Station 2	0.249	−4.358	0.014	0.535	−0.845	0.013	0.002	0.999	−0.001
Station 3	9.012	−4.254	−0.026	0.566	−0.825	−0.001	−0.999	0.013	0.006
Station 4	4.154	4.189	−0.012	0.550	−0.835	0.008	0.002	−0.999	0.001
Station 5	−6.718	4.249	0.029	0.932	0.363	0.002	0.999	−0.004	0.001

**Table 3:** Position and reprojection error of estimated LiDAR position and orientation.

Station Number	Position Error					Reprojection Error (pixels)
	X (m)	Y (m)	Z (m)	Euclidean Error (m)	Error/Distance (%)	
Station 1	0.041	0.022	0.026	0.054	0.36	1.88
Station 2	0.034	0.029	0.025	0.051	1.17	2.00
Station 3	0.009	0.065	0.021	0.069	0.69	3.10
Station 4	0.030	0.067	0.010	0.074	1.25	4.08
Station 5	0.056	0.096	0.030	0.115	1.43	3.61
Mean				0.072	0.98	2.93

**Table 4:** Proposed coarse alignment error and semi-automatic registration error.

Target Number	Errors (m)			Target Number	Errors (m)		
	Proposed Method	Proposed + Fine Registration	Semi-Automatic		Proposed Method	Proposed + Fine Registration	Semi-Automatic
#1	0.084	0.037	0.053	#6	0.191	0.073	0.075
#2	0.108	0.024	0.038	#7	0.210	0.035	0.056
#3	0.077	0.048	0.038	#8	0.077	0.081	0.149
#4	0.058	0.008	0.030	#9	0.158	0.021	0.114
#5	0.194	0.078	0.069	#10	0.085	0.097	0.096
Mean					0.124	0.050	0.072

## Data Availability

Data not available due to Korean government laws (Act on the Establishment and Management of Spatial Data).
